# Trajectories of oral mucosal dryness and multidimensional influencing factors in patients undergoing spine surgery: a prospective longitudinal study

**DOI:** 10.1186/s12903-026-07734-8

**Published:** 2026-02-01

**Authors:** Menghui Gao, Zhongmin Fu, Tao Liao, Peifang Li, Lin Zhang, Jiali Chen, Ning Ning

**Affiliations:** 1 Department of Orthopedic Surgery, West China Hospital, Sichuan University / West China School of Nursing, Sichuan University, No.37, Guoxue Lane, Chengdu, Sichuan Province 610041 People’s Republic of China; 2 Department of Lower Limb Trauma Surgery, Sichuan Province Orthopedic Hospital, Chengdu, Sichuan Province 610041 People’s Republic of China; 3 Department of Neurosurgery, Affiliated Hospital of Zunyi Medical University, Zunyi, Guizhou Province 563000 People’s Republic of China

**Keywords:** Spine surgery, Oral mucosal dryness, Latent growth mixture model, Anxiety, Depression

## Abstract

**Background:**

Oral mucosal dryness during the perioperative period is often underestimated in the postoperative rehabilitation of patients undergoing spine surgery. This study aimed to explore the oral mucosal dryness trajectories and their key influencing factors in these patients.

**Methods:**

This prospective cohort study included 303 patients undergoing spine surgery between February and July 2022. Data on oral mucosal dryness were collected at admission, 2 h, and 6 h post-surgery. Multidimensional factors, including physiological, psychological, and environmental variables, were recorded. Latent Growth Mixture Modeling was used to analyze different trajectories of oral mucosal dryness, and Lasso regression identified significant factors associated with dryness. Multivariate logistic regression was used to investigate the factors influencing each trajectory.

**Results:**

We identified three distinct trajectories of oral mucosal dryness: moderate level increase (38.7%), persistently low level (46.3%), and high-level maintenance (15%). The moderate level increase trajectory was associated with factors including age, prolonged post-anesthesia care unit-to-ward transfer, obesity, antihypertensive use, higher preoperative pain scores, and elevated temperature, while increased environmental humidity and preoperative fluid infusion were associated with reduced risk. The high-level maintenance trajectory was associated with age, body temperature, pain score, plasma drainage, and higher ASA classification, whereas increased environmental humidity was associated with lower risk.

**Conclusion:**

Environmental humidity at 6 h postoperatively and preoperatively pain scores are common factors in oral mucosal dryness and warrant timely attention. Early interventions for high-risk groups at admission may help reduce postoperative oral mucosal dryness, but confirmatory interventional studies are needed.

**Trial registration:**

This trial was registered in the Chinese Clinical Trial Registry (ChiCTR2200064905) on October 21, 2022, retrospective registration.

**Supplementary Information:**

The online version contains supplementary material available at 10.1186/s12903-026-07734-8.

## Introduction

Spine surgery is characterized by significant trauma, prolonged anesthesia duration [1, 2], and preoperative fasting requirements [3], which lead to reduced salivation, decreased oral self-cleansing ability, and, subsequently, issues such as thirst, dry lips, and mucosal damage [4]. Studies have shown that the postoperative thirst incidence rate in perioperative patients is 73.85% [5], and as high as 79.5% in orthopedic patients [6]. Oral mucosal dryness, a significant physiological manifestation of thirst, directly impacts patient comfort and recovery. However, perioperative oral mucosal dryness remained an underappreciated clinical issue in the postoperative rehabilitation of patients undergoing spine surgery. During the perioperative period, healthcare professionals tended to focus more on severe complications such as bleeding, infection, and deep vein thrombosis, lacking a systematic understanding of the management of oral mucosal dryness [7]. Additionally, most healthcare providers lacked knowledge of thirst and standardized perioperative thirst management protocols, leading to an underestimation of the issue [8, 9]. From the patient’s perspective, the traditional notion of “postoperative fasting” prevented them from expressing discomfort related to oral mucosal dryness. According to surveys, only 13%–18% of patients voluntarily report thirst, with most silently enduring it [10]. When patients presented signs of mucosal dryness, such as dry mouth, thick saliva, thick tongue coating, or cracked lips, it indicated that local water loss had reached a severe level [11].

Oral mucosal dryness not only reduces patient comfort but may also lead to complications such as anxiety, aspiration, and oral infections [12] and even increases the risk of postoperative delirium and catheter displacement [13]. In recent years, the Enhanced Recovery After Surgery (ERAS) concept has emphasized improving the patient experience [14]. However, current ERAS guidelines and expert consensus reports lack sufficient attention to risk identification and intervention strategies for oral mucosal dryness in patients undergoing spine surgery. Compared to other elective surgeries, patients undergoing spine surgery face a higher risk of oral mucosal dryness. On the one hand, approximately 85% of spine surgeries required a prone position [15], during which the mouth was exposed to a dry environment for prolonged periods, resulting in increased mucosal moisture evaporation compared to surgeries performed in a supine position. On the other hand, postoperative brace use and pain further limited the patient’s ability to drink independently, exacerbating oral mucosal dryness [16]. There is a lack of specialized research on oral mucosal dryness in spine surgery patients, and existing studies in other fields often focus on static risk factors and single-point assessments of oral mucosal dryness, neglecting the dynamic interactions of multidimensional factors.

Therefore, this study aimed to explore the dynamic changes in oral mucosal dryness and the key influencing factors during the perioperative period in these patients, to identify high-risk groups for thirst in spine surgery, to propose individualized intervention strategies for different trajectory subgroups, to enhance patient comfort, and promote rapid recovery.

## Methods

### Study design and participants

This observational study included patients undergoing spine surgery between February and July 2022. The inclusion criteria were age ≥ 18 years, patients undergoing spine surgery, and patients able to understand and respond to the questionnaire. The exclusion criteria were patients with communication disorders, mental illnesses, swallowing dysfunctions, oral sensory disorders, oral mucosal dryness, patients transferred to other departments after surgery, and patients unable to participate in the survey. After ensuring that participants fully understood the study, they voluntarily signed informed consent and were enrolled. The same surgical team performed all surgeries. This study was approved by the Research Ethics Committee of West China Hospital of Sichuan University (Protocol No. 2022.326), and the clinical trial registration number is ChiCTR2200064905. The reporting quality was assessed using the Strengthening the Reporting of Observational Studies in Epidemiology (STROBE) statement [17].

### Data collection and measurement

#### Dependent variable

The primary outcome was perioperative oral mucosal dryness, assessed using the Objective Oral Mucosa Scale (OOMS) [18]. The scale evaluates lip and oral mucosal dryness: 1 for moist lips and oral cavity, 2 for dry lips and moist oral cavity, 3 for dry lips and dry oral cavity, and 4 for cracked lips and dry oral cavity. Higher scores indicate more dryness. Assessments were made at admission (baseline), 2 h post-surgery, and 6 h post-surgery. Based on a previous study [19], the selection of time points was determined as follows: the average time for patients from anesthesia to ward transfer was about 2 h. At this point, patients were conscious and typically not allowed oral intake, making the 2-hour mark the optimal initial time for ward nurses to assess symptoms. The 6-hour postoperative period represented a critical observation window for surgical patients, during which various symptoms were most prominent.

#### Independent variables

Data were collected through electronic medical records and standardized assessment tools:

- a. *Demographic data*: Gender, age, ethnicity, marital status, education, admission type, height, weight, BMI, body temperature, pulse rate, and respiratory rate.
- b. *Clinical history*: Hypertension, diabetes, antihypertensive medications, antidiabetic medications, preoperative blood glucose, and serum sodium levels.
- c. *Preoperative status*: Preoperative fasting duration, oral carbohydrate administration 2 h preoperatively.

The Huaxi Emotional Distress Index (HEI) was a 9-item questionnaire designed to measure psychological distress [20]. The internal consistency of the HEI was α = 0.918. It was a validated, concise tool used for screening mood disorders and assessing depression, anxiety symptoms, and suicide risk among hospitalized patients in Chinese general hospitals.

Sleep quality was evaluated using the Pittsburgh Sleep Quality Index (PSQI) [21]​​, which was used to assess the quality and patterns of sleep over one month. It included 19 items grouped into seven components: subjective sleep quality, sleep latency, sleep duration, habitual sleep efficiency, sleep disturbances, use of sleep medication, and daytime dysfunction. Higher scores indicated poorer sleep quality.

The Comfort level was measured by the General Comfort Questionnaire (GCQ)​​ [22], which was designed to assess patients’ overall comfort in a hospital setting. The GCQ evaluated physical, emotional, and environmental aspects of comfort, providing a comprehensive assessment of a patient’s comfort level during hospitalization. The GCQ had been validated for use in various healthcare settings and was useful for identifying areas where comfort could be improved to enhance patient recovery.

- d. *Surgical variables*: American Society of Anesthesiologists (ASA) classification, anesthesia type, endotracheal intubation, surgical site, surgery duration, and post-anesthesia care unit (PACU)-to-ward transfer time.
- e. *Fluid volume (mL)*: Daily water intake, preoperative fluid infusion, intraoperative total output, intraoperative urine output, intraoperative blood loss, intraoperative fluid infusion, intraoperative blood transfusion volume, postoperative 6-hour fluid infusion, postoperative 6-hour oral fluid intake, postoperative 6-hour total output, postoperative 6-hour urine output, postoperative 6-hour plasma drainage.
- f. *Dynamic monitoring*: Visual Analog Scale (VAS) scores [23] for pain at admission, 30 min preoperatively, and 2 h, 6 h postoperatively. Ambient temperature and humidity were measured using calibrated hygrothermographs at admission, 30 min preoperatively, 2 and 6 h postoperatively.

### Sample size calculation

The sample size calculation was based on the number of potential variables (39 factors). The sample size was calculated to be 5–10 times the number of independent variables [24], yielding a range of 195–390 cases. Considering a 20% dropout rate, the required sample size was adjusted to 234–468 cases. A total of 319 patients met the inclusion criteria, with 10 patients unable to continue the survey due to surgery suspension and 6 transferred to the ICU. Finally, 303 patients completed the follow-up.

### Statistical analysis

Normally distributed continuous data were expressed as mean and standard deviation (SD), and group comparisons were performed using analysis of variance (ANOVA). Non-normally distributed continuous data were expressed as median (P25, P75), and group comparisons were conducted using the Kruskal-Wallis test. Categorical data were presented as rates, and group comparisons were made using the Chi-square test or Fisher’s exact test. First, we used Mplus 8.7 software to construct a Latent Growth Mixture Model (LGMM) to explore the heterogeneous trajectories of oral mucosal dryness in patients undergoing spine surgery. This is a parametric model suitable for longitudinal data. Unlike traditional models, LGMM identifies latent classes with similar characteristics across the entire population and allows for individual differentiation within the same class, depicting average growth curves. We used three criteria to evaluate model fit and determine the optimal classification: (a) Akaike Information Criterion (AIC), Bayesian Information Criterion (BIC), and sample size-adjusted BIC (aBIC): the lower the value, the better the model fit; (b) Lo-Mendell-Rubin likelihood ratio test (LMR) and Bootstrap likelihood ratio test (BLRT): models with higher p-values show better k-class fit; (c) entropy: ranging from 0 to 1. When this value is closer to 1, the model is sufficiently divided into several classes. Additionally, we ensured that the proportion of each class in the final model was greater than 5% [25–28]. Next, Lasso regression analysis was performed using STATA 17 software to select the independent variables. Finally, multivariate logistic regression analysis was conducted using R 4.4.2 software, with the glm function to explore the influencing factors of different oral mucosal dryness trajectories. We checked for multicollinearity of the independent variables (by calculating the variance inflation factor, VIF) to ensure model stability. Trajectory plots were generated using the ggplot2 package in R, and Lasso regression coefficient plots were created using STATA 17 software. The significance level for all statistical tests was set at two-tailed 0.05.

## Results

### Latent heterogeneity trajectories of oral mucosal dryness

**Fig. 1:**
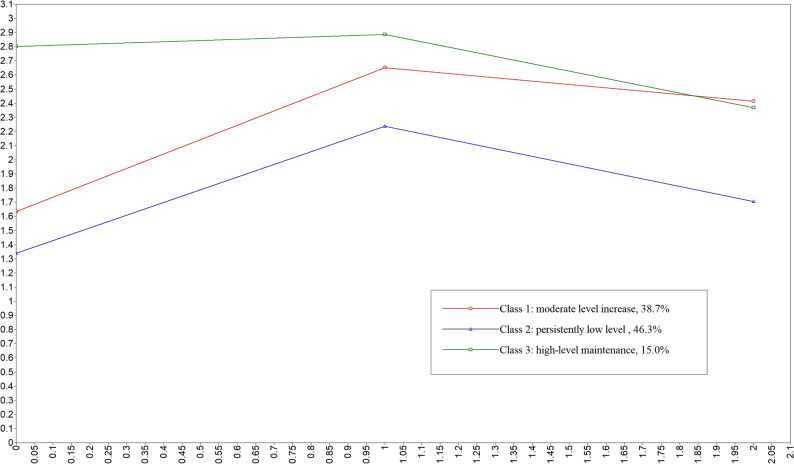
Trajectories of oral mucosal dryness in patients undergoing spine surgery

To capture the different patterns of change in oral mucosal dryness over time, we employed LGMM. As shown in Table 1, the three-class model (C3) provided the best balance of fit and parsimony. Key indicators for this choice included: a statistically significant improvement in fit over the two-class model (as indicated by both the LMR and BLRT p-values < 0.001), a high entropy value (0.858), suggesting clear class separation, and reasonable class probabilities (each > 5%). Although the four-class model (C4) had slightly better AIC/BIC values, its LMR test was non-significant (*p* = 0.356), and it included one very small class (4.6%), which was less clinically meaningful. Therefore, the three-class solution was selected. Based on this model, Fig. 1 shows three categories of oral mucosal dryness in patients undergoing spine surgery: Class 1, moderate level increase (38.7%); Class 2, persistently low level (46.3%); Class 3, high-level maintenance (15%).

**Table 1:** Latent growth mixture model fit information

Model	Log(L)	AIC	BIC	aBIC	Entropy	LMR(*p*)	BLRT(*p*)	Category Probability
C1	-1026.941	2063.882	2082.45	2066.593				
C2	-1012.466	2040.933	2070.643	2045.271	0.617	0.0051	< 0.001	77.2/22.8
C3	-1009.544	2041.088	2081.939	2047.053	0.858	< 0.001	< 0.001	38.7/46.3/15.0
C4	-825.927	1679.853	1731.846	1687.445	0.871	0.356	0.001	4.6/21.5/20.1/53.8

*Log(L)* Log-Likelihood, *AIC* Akaike Information Criterion, *BIC* Bayesian Information Criterion, *aBIC* Adjusted Bayesian Information Criterion, *LMR* Lo-Mendell-Rubin Likelihood Ratio Test, *BLRT* Bootstrap Likelihood Ratio Test

## Results of Lasso regression analysis

**Fig. 2:**
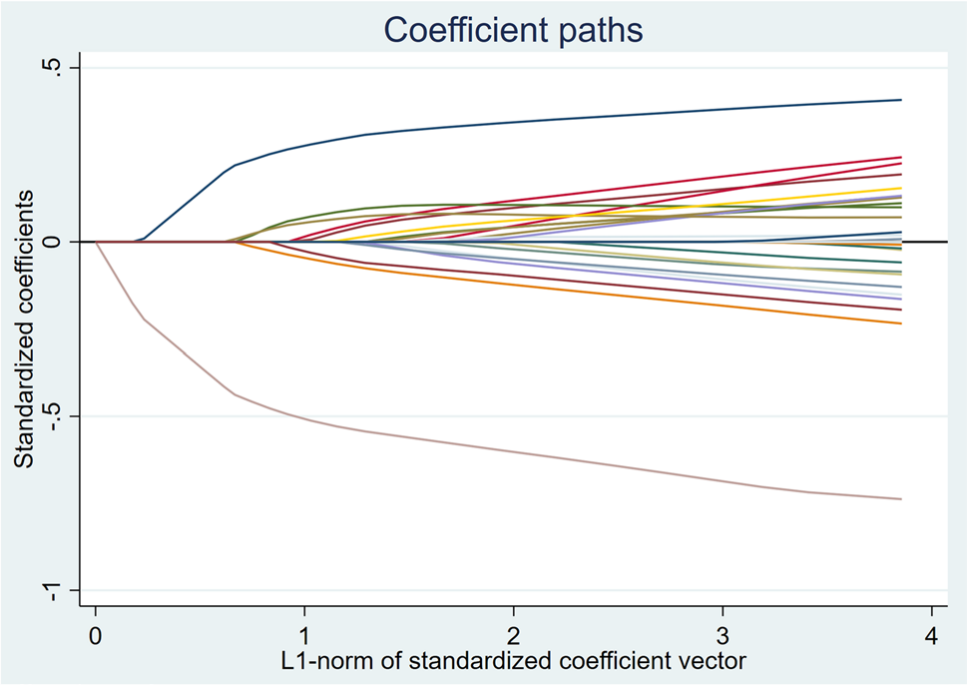
Lasso regression coefficients for significant predictors of oral mucosal dryness trajectories in patients undergoing spine surgery

Lasso regression analysis used cross-validation to determine the optimal λ value (λ = 0.021), selecting 21 significant predictors (Table 2; Fig. 2). The model provided the best fit at this λ value and effectively reduced overfitting.

**Table 2:** Most influential predictors selected by the best-fitting Lasso logistic model

Most influential positive (higher risk) predictors	Coefficient	Most influential negative (lower risk) predictors	Coefficient
Age	0.3705	Postoperative 6 h ambient humidity	-0.6622
Body temperature	0.1683	Education	-0.1668
HEI	0.1365	Surgical site	-0.1362
VAS 30 min before surgery	0.1184	Antihypertensive medications	-0.0916
VAS on admission	0.1034	Daily water intake	-0.0821
Postoperative 6-hour fluid infusion volume	0.1029	Preoperative fluid infusion volume	-0.0733
PACU-to-ward transfer time	0.0951	GCQ score	-0.0534
ASA classification	0.0734	Oral carbohydrate administration 2 h preoperatively	-0.0200
Nation	0.0717		
Weight	0.0695		
Postoperative 6-hour plasma drainage volume	0.0638		
VAS 2 h postoperatively	0.0454		
Intraoperative blood loss	0.0150		

*GCQ* general comfort questionnaire scores​, *VAS* ​​Visual analog Scale​, *HEI* Huaxi Emotional-Distress index

### Baseline characteristics

The baseline comparisons of the 21 predictor variables among the three groups are presented in Table 3, while the baseline comparisons of additional variables are provided in the supplementary material (Table S1). Class 3 patients were older (60.70 ± 16.31) and had higher ASA III classification proportions compared to other groups (*P* < 0.05). In contrast, Class 1 patients had a higher prevalence of hypertension (35.42%) and antihypertensive medication use (34.38%) (*P* = 0.023), while Class 2 patients were the youngest (49.98 ± 15.00). Significant intergroup differences in environmental humidity were observed a 6 h post-surgery (*P* < 0.001).

**Table 3:** Baseline characteristics of 21 predictor variables among the three groups

Variables	Total (*n* = 303)	Class 1 (*n* = 96)	Class 2 (*n* = 167)	Class 3 (*n* = 40)	χ²/F/Fisher	*P*
Age (year), Mean ± SD	53.75 ± 15.40	57.41 ± 13.88	49.98 ± 15.00	60.70 ± 16.31	12.70	**< 0.001**
Nation, *n* (%)					-	0.146
​​Han	288 (95.05)	87 (90.62)	162 (97.01)	39 (97.50)		
Tibetan​	5 (1.65)	4 (4.17)	1 (0.60)	0 (0.00)		
Yi	2 (0.66)	2 (2.08)	0 (0.00)	0 (0.00)		
​​Others​	8 (2.64)	3 (3.12)	4 (2.40)	1 (2.50)		
Antihypertensive medications​, *n *(%)					7.58	**0.023**
Yes	75 (24.75)	33 (34.38)	32 (19.16)	10 (25.00)		
No	228 (75.25)	63 (65.62)	135 (80.84)	30 (75.00)		
Oral carbohydrate administration 2 h preoperatively, *n *(%)					1.38	0.502
Yes	242 (79.87)	80 (83.33)	132 (79.04)	30 (75.00)		
No	61 (20.13)	16 (16.67)	35 (20.96)	10 (25.00)		
ASA classification, *n *(%)					-	**< 0.001**
Ⅰ	2 (0.66)	0 (0.00)	2 (1.20)	0 (0.00)		
Ⅱ	233 (76.90)	72 (75.00)	139 (83.23)	22 (55.00)		
Ⅲ	68 (22.44)	24 (25.00)	26 (15.57)	18 (45.00)		
​​Surgical site, *n *(%)					-	0.098*
Neck	90 (29.70)	30 (31.25)	43 (25.75)	17 (42.50)		
Thoracic	24 (7.92)	5 (5.21)	13 (7.78)	6 (15.00)		
Lumbar	168 (55.45)	57 (59.38)	96 (57.49)	15 (37.50)		
Multiple sites	21 (6.93)	2 (2.16)	15(8.98)	2 (5.00)		
Education, *n *(%)					16.26	0.092
Below the primary school	21 (6.93)	11 (11.46)	7 (4.19)	3 (7.50)		
Primary school	52 (17.16)	23 (23.96)	20 (11.98)	9 (22.50)		
Junior high school	76 (25.08)	20 (20.83)	46 (27.54)	10 (25.00)		
Senior high school	60 (19.80)	16 (16.67)	34 (20.36)	10 (25.00)		
Associate degree	50 (16.50)	14 (14.58)	31 (18.56)	5 (12.50)		
Bachelor’s degree or higher	44 (14.52)	12 (12.50)	29 (17.37)	3 (7.50)		
​​Body Temperature​, Mean ± SD	36.46 ± 0.21	36.45 ± 0.22	36.45 ± 0.21	36.49 ± 0.16	0.60	0.55
Weight, Mean ± SD	64.38 ± 11.24	65.44 ± 11.07	64.20 ± 11.48	62.57 ± 10.57	0.96	0.383
Postoperative 6 h ambient humidity(%), Mean ± SD	56.93 ± 11.82	53.09 ± 10.86	60.74 ± 11.09	50.27 ± 11.34	22.96	**< 0.001**
​​HEI, M (Q₁, Q₃)	0.00 (0.00, 2.00)	0.00 (0.00,2.00)	0.00 (0.00,1.50)	0.00 (0.00,2.00)	2.02#	0.365
GCQ scores, Mean ± SD	48.36 ± 9.71	48.06 ± 9.87	48.89 ± 9.19	46.88 ± 11.37	0.76	0.467
PACU-to-ward transfer time (h)​​, Mean ± SD	1.10 ± 0.73	1.23 ± 0.77	1.01 ± 0.70	1.17 ± 0.71	2.95	0.054
VAS on admission, M (Q₁, Q₃)	0.00 (0.00, 3.00)	1.00 (0.00,3.00)	0.00 (0.00,3.00)	0.00 (0.00,2.25)	1.04#	0.595
VAS 30 min before surgery, M (Q₁, Q₃)	0.00 (0.00, 0.00)	0.00 (0.00,0.00)	0.00 (0.00,0.00)	0.00 (0.00,0.25)	0.07#	0.567
VAS 2 h postoperatively, M (Q₁, Q₃)	2.00 (0.00, 3.00)	2.00 (0.00,3.00)	2.00 (0.00,3.00)	0.00 (0.00,3.00)	1.57#	0.456
Daily water intake (mL), M (Q₁, Q₃)	1100.00 (600.00, 1600.00)	1000.00 (500.00,1600.00)	1200.00 (735.00,1675.00)	1000.00 (537.50,1300.00)	3.22#	0.200
Preoperative fluid infusion volume (mL), M (Q₁, Q₃)	400.00 (200.00, 500.00)	400.00 (300.00,512.50)	400.00 (200.00,500.00)	300.00 (200.00,500.00)	7.30#	**0.026**
Intraoperative blood loss (mL)​, M (Q₁, Q₃)	100.00 (15.00, 200.00)	100.00 (20.00,300.00)	50.00 (10.00,200.00)	100.00 (0.00,162.50)	3.25#	0.197
Postoperative 6-hour fluid infusion volume (mL)​, M (Q₁, Q₃)	600.00 (100.00, 900.00)	665.00 (200.00,1013.75)	600.00 (100.00,800.00)	600.00 (100.00,1000.00)	5.46#	0.065
postoperative 6-hour plasma drainage volume (mL), M (Q₁, Q₃)	0.00 (0.00, 80.00)	0.00 (0.00,72.50)	0.00 (0.00,80.00)	0.00 (0.00,85.00)	0.48#	0.788

*F *ANOVA,* # *Kruskal-waills test,* χ² *Chi-square test,* - *Fisher exact*, SD *standard deviation,* M *Median,* Q₁ *1st Quartile,* Q₃ *3st Quartile,* GCQ *General Comfort Questionnaire scores*​, VAS *Visual Analog Scale*​, HEI *Huaxi Emotional Distress Index

### Factors influencing different oral mucosal dryness trajectories

Multivariate logistic regression analysis revealed differentiated predictive factors for the trajectories of oral mucosal dryness in patients undergoing spine surgery (Table 4).

**Table 4:** Analysis of influencing factors on oral mucosal dryness trajectory in spinal surgery patients​​

Variable	OR (95% CI)	*P*-value
Class 1 vs. Class 2		
Postoperative 6 h ambient humidity (%)	0.93 (0.90–0.96)	< 0.001
Age (years)	1.03 (1.01–1.06)	0.013
PACU-to-ward transfer time (h)	1.62 (1.04–2.55)	0.035
Weight (kg)	1.04 (1.00-1.08)	0.038
Antihypertensive medications		
Yes	2.45(1.13–5.31)	0.024
No	-	-
Body Temperature (°C)	1.35 (1.01–1.80)	0.042
Preoperative fluid infusion volume (mL)	0.87 (0.35–0.98)	0.043
VAS 30 min before surgery	1.41 (1.06–1.87)	0.018
Class 3 vs. Class 2		
Postoperative 6 h ambient humidity (%)	0.88 (0.84–0.92)	< 0.001
Age (years)	1.04 (1.01–1.08)	0.026
Body Temperature (°C)	1.97 (1.31–2.96)	0.001
VAS 30 min before surgery	1.77 (1.17–2.67)	0.007
Postoperative 6-hour plasma drainage volume (mL)	1.01 (1.00-1.01)	0.012
HEI	1.22 (1.01–1.47)	0.037
ASA classification		
Ⅰ	0.01 (0.00-0.02)	0.002
Ⅱ	0.26(0.08–0.81)	0.020
Ⅲ	-	-

*VAS* visual analog Scale, *HEI* Huaxi Emotional-Distress index

### Class 1 vs class 2

Compared to Class 2, independent risk factors for the moderate level increase trajectory (38.7%) included advanced age (OR = 1.03), prolonged PACU-to-ward transfer time (OR = 1.62), higher weight (OR = 1.04), use of antihypertensive medications (OR = 2.45), elevated body temperature (OR = 1.35), and higher preoperative VAS pain scores 30 min before surgery (OR = 1.41). Increased postoperative 6-hour environmental humidity (OR = 0.93) and preoperative fluid infusion volume (OR = 0.87) were protective factors for the moderate level increase trajectory.

### Class 3 vs class 2

Compared to the persistently low level trajectory (Class 2), each 1% increase in postoperative 6-hour environmental humidity reduced the risk of the high-level maintenance trajectory by 12% (OR = 0.88). Additionally, advanced age (OR = 1.04), higher preoperative VAS pain scores (OR = 1.77), increased body temperature (OR = 1.97), increased postoperative 6-hour plasma drainage (OR = 1.01), and higher HEI scores (OR = 1.22) significantly increased the risk of high-level maintenance oral mucosal dryness. ASA classification showed that the risk of high-level maintenance oral mucosal dryness was 99% lower in Class I compared to Class III patients (OR = 0.01, *P* = 0.002) and 74% lower in Class II patients (OR = 0.26, *P* = 0.020).

## Discussion

The study found that oral mucosal dryness in these patients exhibited three typical trajectories: moderate level increase, persistently low level, and high-level maintenance. The moderate level increase and high-level maintenance types together accounted for more than half of the cases. Factors associated with the moderate level increase trajectory included older age, obesity, elevated body temperature, lower preoperative fluid infusion, decreased ambient humidity at 6 h post-surgery, prolonged PACU-to-ward transfer time, use of antihypertensive medications, and higher VAS pain scores 30 min before surgery. The high-level maintenance trajectory was associated with decreased postoperative ambient humidity, older age, elevated body temperature, higher preoperative VAS pain scores, increased postoperative plasma drainage, higher ASA classification, and negative emotional states.

Environmental humidity, age, body temperature, and higher VAS pain scores 30 min before surgery were common influencing factors for both the moderate level increase and high-level maintenance trajectories of oral mucosal dryness. Notably, the change in environmental humidity at 6 h post-surgery directly affected oral hydration. Low humidity environments accelerate the evaporation of saliva, decreasing the moisture of the oral mucosa and worsening the sensation of dryness [29]. Consistent with other studies, low humidity was identified as a significant external factor contributing to oral mucosal dryness [30, 31]. The lack of significant effects of preoperative and 2-hour postoperative humidity changes on oral mucosal dryness may have been related to the following reasons: preoperatively, patients were generally in a stable physiological state and could drink independently, so humidity changes had minimal impact; at 2 h post-surgery, patients were still in the anesthesia recovery phase, and dryness was more influenced by anesthetic drugs, fluid loss, and postoperative physiological changes rather than environmental humidity. However, by 6 h post-surgery, as patients’ anesthesia state recovered, environmental humidity became a more significant factor affecting oral hydration. Therefore, clinical care should have particularly focused on humidity management during this key recovery period, optimizing oral care for patients. Although current studies suggested that using humidification devices in hospital rooms could effectively improve staff comfort, the benefits for patients may diminish [32]. Future studies could further determine the optimal humidity range and explore the interaction between humidity and other environmental factors (such as temperature and air flow) to optimize hospital room settings under multi-factor conditions, thus reducing the incidence of oral mucosal dryness. Additionally, the long-term effects of humidity regulation and its sustained impact on patient quality of life should be further validated through long-term follow-up research to provide more systematic strategies for clinical humidity intervention.

Age has been confirmed as an important predictor of oral mucosal dryness [33, 34]. As individuals age, the decline in salivary gland function and hydration capacity increases the likelihood of oral mucosal dryness during the postoperative recovery phase, particularly in elderly patients [34]. Additionally, aging is associated with decreased kidney function and diminished thirst sensation, leading many elderly patients to refrain from drinking, thereby exacerbating oral mucosal dryness [18, 35]. Therefore, it is important to focus on early recognition and intervention for oral mucosal dryness in elderly patients during postoperative care. Previous studies have shown that elevated body temperature is positively correlated with oral mucosal dryness [36, 37]. Increased temperature accelerates basal metabolic consumption and stimulates the thirst center to induce oral mucosal dryness [38]. This suggests that clinicians should closely monitor fluid intake (either orally or through infusion) and oral mucosal dryness symptoms in patients with moderate to high body temperature and take timely nursing measures. The preoperative pain score at 30 min was found to be related to oral mucosal dryness, whereas pain scores at admission, 2 h, and 6 h post-surgery did not significantly affect oral mucosal dryness. This phenomenon may be due to the patient’s stress response, anesthetic drugs, and analgesic medications. Various hormones regulate pain production, including adrenocorticotropic hormone (ACTH), cortisol, and hormones involved in the renin-angiotensin-aldosterone system [39, 40]. The 30-minute preoperative period is a critical time when pain activates the stress response, reducing salivation and exacerbating oral mucosal dryness symptoms [41]. In contrast, patients at 2 and 6 h post-surgery typically receive analgesic management, and the metabolic effects of anesthetic and analgesic medications help alleviate pain and stress responses, thus reducing the perception of dryness. Therefore, the pain score at 30 min before surgery reflects a higher stress level and pain sensitivity, making pain management during this period particularly important for preventing and managing oral mucosal dryness. Non-pharmacological interventions such as distraction techniques, mindfulness meditation, or relaxation training can be used to alleviate preoperative pain and improve oral mucosal dryness during early postoperative recovery [42].

The study found that increased preoperative fluid infusion was a protective factor for the moderate level increase trajectory of oral mucosal dryness, indicating that adequate preoperative fluid intake helps maintain normal blood volume and relieve oral mucosal dryness. Consistent with this, ERAS guidelines indicate that prolonged fasting (> 12 h) can lead to hypovolemia and increased metabolic stress, while the latest guidelines from the American Society of Anesthesiologists allow a 2-hour preoperative fasting period to prevent dehydration [43]. Other studies show that consuming 250 ml of 5% glucose solution 2–3 h before surgery can reduce oral mucosal dryness without increasing gastric residual volume or perioperative complications [44]. Therefore, preoperative nutritional drinks (carbohydrate drinks) and fluid supplementation should be provided to minimize complications and the sensation of oral mucosal dryness in spine surgery patients.

Obese patients undergoing spine surgery are more likely to exhibit moderate level increase oral mucosal dryness, which may involve multiple physiological mechanisms. Obese individuals may experience abnormal release of vasopressin, a hormone induced by dehydration that plays an important role in maintaining fluid homeostasis by promoting water reabsorption in the kidneys and concentrating urine [45]. In obese individuals, higher fat storage leads to increased leptin secretion, which correlates with fat storage [46]. Leptin increased the expression of vasopressin mRNA [47]. Additionally, changes in hormone levels in obese individuals may further affect the release of vasopressin [48]. These hormonal interactions may exacerbate fluid balance disturbances, negatively affecting hydration and leading to symptoms such as oral mucosal dryness. Therefore, fluid management in obese patients during the postoperative recovery phase should pay special attention to hormone levels to alleviate oral mucosal dryness symptoms. The use of antihypertensive medications has been proven to be a significant cause of oral mucosal dryness [49–51]. Studies have shown that the highest incidence of xerostomia in the elderly occurs with the use of calcium channel blockers (31.1%) and diuretics (26.8%) [50]. Furosemide reduces electrolyte output, especially potassium and chloride in submandibular and sublingual gland secretions [52, 53]. Calcium channel blockers reduce H2O secretion by blocking Ca2 + channels, leading to oral mucosal dryness [54]. Therefore, for patients using diuretics and calcium channel blockers, preoperative and postoperative management should be strengthened. Fluid intake should be increased preoperatively, and humidity control should be enhanced in the early postoperative recovery period to prevent worsening of oral mucosal dryness due to medication effects. The prolonged PACU-to-ward transfer time was also associated with the moderate level increase trajectory of oral mucosal dryness. During the transfer to the ward, inadequate fluid infusion and prolonged exposure to low humidity environments may be major contributors to oral mucosal dryness. Early assessment of oral mucosal dryness [55] and proactive oral moisture management, such as avoiding mouth breathing and using oral moisturizing devices during transfer, should be prioritized to reduce oral mucosal dryness.

In contrast, the occurrence of high-level maintenance oral mucosal dryness was closely related to postoperative 6-hour plasma drainage volume and higher ASA classification. The relationship between postoperative 6-hour plasma drainage and oral mucosal dryness may reflect early fluid balance issues. Increased plasma drainage suggests excessive fluid loss post-surgery, which may be related to improper fluid management. Therefore, reasonable fluid management, close monitoring of plasma drainage, and maintaining fluid balance during the early postoperative recovery are crucial to reducing oral mucosal dryness. Additionally, the ASA classification, which assesses preoperative health status, shows that higher ASA levels indicate more complex surgeries, requiring more anesthetic drugs, longer surgery duration, and larger incisions [56, 57], all of which increase the risk of early postoperative oral mucosal dryness.

Interestingly, patients with preoperative psychological distress were also more likely to experience high-level oral mucosal dryness, suggesting an interaction between psychological stress and thirst perception. Consistent with previous studies, anxiety and depression may exacerbate postoperative discomfort, including oral mucosal dryness [58, 59]. Psychological distress, such as anxiety and depression, often accompanies the body’s stress response, which may affect hydration and oral moisture through various mechanisms. Studies have shown that increased anxiety and stress can raise levels of stress hormones, such as cortisol [60, 61], which not only affect renal water reabsorption but may also reduce salivation, thereby worsening oral mucosal dryness [62, 63]. Compared to physiological factors, psychological stress is often multi-dimensional and may interact with physiological responses through complex neuroendocrine pathways, leading to enhanced dryness [64]. It was noted that factors contributing to anxiety levels in patients awaiting elective surgery included concerns about family, fear of complications, surgical outcomes, and postoperative pain [65]. Therefore, clinical care should particularly focus on the psychological state of preoperative patients, especially those with anxiety, depression, or other emotional distress, and utilize psychological interventions, mindfulness meditation, or relaxation techniques [66] to help alleviate psychological stress. This may help reduce the symptoms of persistent oral mucosal dryness and accelerate the early postoperative recovery process.

### Study limitations and strengths

This study has several limitations. First, although the study was based on data from 303 patients undergoing spine surgery, representing a certain population, the heterogeneity of spine surgery, which includes patients of various ages and pathological conditions, limited subgroup analysis due to relatively small sample sizes. Second, the follow-up period was restricted to the first 6 h postoperatively. While this captures early critical changes, oral mucosal dryness may change beyond this time point, and the lack of longer-term data limits our understanding of its full trajectory. The long-term variation in oral mucosal dryness may be influenced by persistent factors such as long-term medication use, fluid management, and dietary changes. Finally, although the study found a strong relationship between psychological distress (such as anxiety and depression) and oral mucosal dryness, the relationship is complex, and given the observational design, causality cannot be determined. The multidimensional and dynamic nature of psychological states makes it difficult to attribute this factor to a single cause. Future studies could further explore the interaction mechanisms, including by collecting data on various hormone levels.

Despite these limitations, this study has several strengths. It is the first to reveal the three dynamic trajectories of oral mucosal dryness in patients undergoing spine surgery, and it deeply analyzes the physiological, psychological, and environmental factors that influence oral mucosal dryness in this population, providing clinically meaningful and actionable results. Furthermore, we selected multiple important time points (e.g., 30 min preoperatively, 2 h post-surgery, 6 h post-surgery) to identify key events affecting oral mucosal dryness. This offers a more detailed time framework for future research and helps pinpoint critical moments requiring attention. Finally, based on the key influencing factors identified, we have proposed clinical intervention strategies, including optimizing preoperative fluid management, improving postoperative humidity control, enhancing pain management, and providing psychological interventions, offering practical guidelines for clinical care.

## Conclusion

This study was the first to reveal the three dynamic trajectories of oral mucosal dryness in patients undergoing spine surgery: moderate level increase, persistently low level, and high-level maintenance. Environmental humidity, fluid management, psychological status, and preoperative pain control may play key roles to prevent oral mucosal dryness in these patients. Clinical care should focus particularly on the early postoperative period, especially humidity management at 6 h post-surgery. Future research should explore the optimal humidity range and the complex interaction mechanisms between oral mucosal dryness and psychological distress to improve patient comfort and enhance postoperative recovery.

## Supplementary Information

Supplementary Material 1.

## Data Availability

The data that support the findings of this study are available from the corresponding author upon reasonable request.
