# Supplementary material for: Trajectories of oral mucosal dryness and multidimensional influencing factors in patients undergoing spine surgery: a prospective longitudinal study
Source: BMC Oral Health. 2026 Feb 1;26:427. doi: 10.1186/s12903-026-07734-8 (PMC12955227; doi:10.1186/s12903-026-07734-8)
Supplement: Supplementary file 1 — Supplementary Material 1. [file 12903_2026_7734_MOESM1_ESM.docx]

**Supplementary Material**

**Table S1. Baseline characteristics (**complete data**)**

| Variables | Total (n = 303) | Class 1  (n = 96) | Class 2  (n = 167) | Class 3  (n = 40) | *χ²*/F/Fisher | *P* |
| --- | --- | --- | --- | --- | --- | --- |
| **Gender, n(%)** |  |  |  |  | 0.40 | 0.818 |
| Female | 142 (46.86) | 43 (44.79) | 81 (48.50) | 18 (45.00) |  |  |
| Male | 161 (53.14) | 53 (55.21) | 86 (51.50) | 22 (55.00) |  |  |
| **Age, Mean ± SD** | 53.75 ± 15.40 | 57.41 ± 13.88 | 49.98 ± 15.00 | 60.70 ± 16.31 | 12.70 | **<.001** |
| **Nation, n(%)** |  |  |  |  | - | 0.146 |
| ​​Han | 288 (95.05) | 87 (90.62) | 162 (97.01) | 39 (97.50) |  |  |
| Tibetan​ | 5 (1.65) | 4 (4.17) | 1 (0.60) | 0 (0.00) |  |  |
| Yi | 2 (0.66) | 2 (2.08) | 0 (0.00) | 0 (0.00) |  |  |
| ​​Others​ | 8 (2.64) | 3 (3.12) | 4 (2.40) | 1 (2.50) |  |  |
| **Marital status, n(%)** |  |  |  |  | - | **0.017** |
| Unmarried | 25 (8.25) | 4 (4.17) | 18 (10.78) | 3 (7.50) |  |  |
| Married | 259 (85.48) | 84 (87.50) | 143 (85.63) | 32 (80.00) |  |  |
| Divorced | 8 (2.64) | 5 (5.21) | 3 (1.80) | 0 (0.00) |  |  |
| Widowed​ | 11 (3.63) | 3 (3.12) | 3 (1.80) | 5 (12.50) |  |  |
| **Admission type, n(%)** |  |  |  |  | 7.69 | **0.021** |
| Elective admission | 270 (89.11) | 90 (93.75) | 149 (89.22) | 31 (77.50) |  |  |
| Emergency admission​ | 33 (10.89) | 6 (6.25) | 18 (10.78) | 9 (22.50) |  |  |
| **Hypertension, n(%)** |  |  |  |  | 8.12 | **0.017** |
| Yes | 76 (25.08) | 34 (35.42) | 33 (19.76) | 9 (22.50) |  |  |
| No | 227 (74.92) | 62 (64.58) | 134 (80.24) | 31 (77.50) |  |  |
| **Diabetes, n(%)** |  |  |  |  | 0.87 | 0.646 |
| Yes | 25 (8.25) | 10 (10.42) | 12 (7.19) | 3 (7.50) |  |  |
| No | 278 (91.75) | 86 (89.58) | 155 (92.81) | 37 (92.50) |  |  |
| **Preoperative blood glucose, n(%)** |  |  |  |  | - | **0.021** |
| Below normal range | 3 (0.99) | 1 (1.04) | 1 (0.60) | 1 (2.50) |  |  |
| Within normal range | 232 (76.57) | 74 (77.08) | 135 (80.84) | 23 (57.50) |  |  |
| Above normal range | 68 (22.44) | 21 (21.88) | 31 (18.56) | 16 (40.00) |  |  |
| ​​**Preoperative serum sodium​, n(%)** |  |  |  |  | - | 0.863 |
| Below normal range | 14 (4.62) | 5 (5.21) | 7 (4.19) | 2 (5.00) |  |  |
| Within normal range | 289 (95.38) | 91 (94.79) | 160 (95.81) | 38 (95.00) |  |  |
| Antihypertensive **medications​, n(%)** |  |  |  |  | 7.58 | **0.023** |
| Yes | 75 (24.75) | 33 (34.38) | 32 (19.16) | 10 (25.00) |  |  |
| No | 228 (75.25) | 63 (65.62) | 135 (80.84) | 30 (75.00) |  |  |
| **Antidiabetic medications​, n(%)** |  |  |  |  | 0.59 | 0.743 |
| Yes | 22 (7.26) | 6 (6.25) | 12 (7.19) | 4 (10.00) |  |  |
| No | 281 (92.74) | 90 (93.75) | 155 (92.81) | 36 (90.00) |  |  |
| **Oral carbohydrate administration 2 hours preoperatively, n(%)** |  |  |  |  | 1.38 | 0.502 |
| Yes | 242 (79.87) | 80 (83.33) | 132 (79.04) | 30 (75.00) |  |  |
| No | 61 (20.13) | 16 (16.67) | 35 (20.96) | 10 (25.00) |  |  |
| **ASA classification , n(%)** |  |  |  |  | - | **<.001** |
| Ⅰ | 2 (0.66) | 0 (0.00) | 2 (1.20) | 0 (0.00) |  |  |
| Ⅱ | 233 (76.90) | 72 (75.00) | 139 (83.23) | 22 (55.00) |  |  |
| Ⅲ | 68 (22.44) | 24 (25.00) | 26 (15.57) | 18 (45.00) |  |  |
| **​anesthesia type​​, n(%)** |  |  |  |  | - | 0.067 |
| General Anesthesia | 297 (98.02) | 96 (100.00) | 164 (98.20) | 37 (92.50) |  |  |
| Neuraxial Anesthesia | 2 (0.66) | 0 (0.00) | 1 (0.60) | 1 (2.50) |  |  |
| Local Anesthesia | 4 (1.32) | 0 (0.00) | 2 (1.20) | 2 (5.00) |  |  |
| **Endotracheal intubation, n(%)** |  |  |  |  | - | **0.047** |
| Yes | 300 (99.01) | 96 (100.00) | 166 (99.40) | 38 (95.00) |  |  |
| No | 3 (0.99) | 0 (0.00) | 1 (0.60) | 2 (5.00) |  |  |
| ​​**Surgical site, n(%)** |  |  |  |  | - | 0.098* |
| Neck | 90 (29.70) | 30 (31.25) | 43 (25.75) | 17 (42.50) |  |  |
| Thoracic | 24 (7.92) | 5 (5.21) | 13 (7.78) | 6 (15.00) |  |  |
| Lumbar | 168 (55.45) | 57 (59.38) | 96 (57.49) | 15 (37.50) |  |  |
| Multiple sites | 21 (6.93) | 2 (2.16) | 15(8.98) | 2 (5.00) |  |  |
| **Education, n(%)** |  |  |  |  | 16.26 | 0.092 |
| Below the primary school | 21 (6.93) | 11 (11.46) | 7 (4.19) | 3 (7.50) |  |  |
| Primary school | 52 (17.16) | 23 (23.96) | 20 (11.98) | 9 (22.50) |  |  |
| Junior high school | 76 (25.08) | 20 (20.83) | 46 (27.54) | 10 (25.00) |  |  |
| Senior high school | 60 (19.80) | 16 (16.67) | 34 (20.36) | 10 (25.00) |  |  |
| Associate degree | 50 (16.50) | 14 (14.58) | 31 (18.56) | 5 (12.50) |  |  |
| Bachelor's degree or higher | 44 (14.52) | 12 (12.50) | 29 (17.37) | 3 (7.50) |  |  |
| ​​**Body Temperature​, Mean ± SD** | 36.46 ± 0.21 | 36.45 ± 0.22 | 36.45 ± 0.21 | 36.49 ± 0.16 | 0.60 | 0.55 |
| ​​Pulse Rate, Mean ± SD | 80.94 ± 13.06 | 80.21 ± 13.40 | 81.16 ± 13.22 | 81.75 ± 11.66 | 0.25 | 0.779 |
| ​​Respiratory Rate​Mean ± SD | 19.32 ± 1.04 | 19.35 ± 1.26 | 19.26 ± 0.88 | 19.48 ± 1.04 | 0.80 | 0.451 |
| Height, Mean ± SD | 163.45 ± 8.49 | 164.12 ± 8.30 | 163.43 ± 8.78 | 161.90 ± 7.66 | 0.97 | 0.38 |
| Weight, Mean ± SD | 64.38 ± 11.24 | 65.44 ± 11.07 | 64.20 ± 11.48 | 62.57 ± 10.57 | 0.96 | 0.383 |
| BMI, Mean ± SD | 24.00 ± 3.14 | 24.19 ± 3.03 | 23.94 ± 3.27 | 23.77 ± 2.94 | 0.32 | 0.726 |
| Ambient temperature on admission, Mean ± SD | 24.90 ± 1.11 | 24.89 ± 1.18 | 24.84 ± 1.07 | 25.17 ± 1.08 | 1.49 | 0.227 |
| Ambient humidity on admission, Mean ± SD | 54.91 ± 11.94 | 51.33 ± 10.41 | 57.84 ± 12.03 | 51.27 ± 11.96 | 12.01 | **<.001** |
| Preoperative ambient temperature, Mean ± SD | 24.47 ± 1.50 | 24.39 ± 1.38 | 24.40 ± 1.32 | 24.96 ± 2.23 | 2.48 | 0.085 |
| Preoperative ambient humidity, Mean ± SD | 60.45 ± 12.83 | 56.75 ± 12.39 | 63.86 ± 12.35 | 55.08 ± 11.78 | 14.59 | **<.001** |
| Postoperative 2h ambient temperature, Mean ± SD | 24.81 ± 1.25 | 24.72 ± 1.30 | 24.76 ± 1.15 | 25.21 ± 1.43 | 2.49 | 0.084 |
| Postoperative 2h ambient humidity, Mean ± SD | 55.61 ± 11.81 | 51.62 ± 10.32 | 59.14 ± 11.56 | 50.45 ± 11.51 | 18.68 | **<.001** |
| Postoperative 6h ambient temperature, Mean ± SD | 24.63 ± 1.23 | 24.55 ± 1.27 | 24.54 ± 1.14 | 25.20 ± 1.37 | 5.15 | **0.006** |
| Postoperative 6h ambient humidity, Mean ± SD | 56.93 ± 11.82 | 53.09 ± 10.86 | 60.74 ± 11.09 | 50.27 ± 11.34 | 22.96 | **<.001** |
| ​​HEI, M (Q₁, Q₃) | 0.00 (0.00, 2.00) | 0.00 (0.00,2.00) | 0.00 (0.00,1.50) | 0.00 (0.00,2.00) | 2.02# | 0.365 |
| PSQI, M (Q₁, Q₃) | 4.00 (2.00, 7.00) | 5.00 (2.00,7.00) | 4.00 (2.00,7.50) | 5.00 (2.75,8.00) | 1.54# | 0.464 |
| GCQ scores, Mean ± SD | 48.36 ± 9.71 | 48.06 ± 9.87 | 48.89 ± 9.19 | 46.88 ± 11.37 | 0.76 | 0.467 |
| Preoperative fasting duration for liquids​, Mean ± SD | 9.80 ± 3.87 | 9.83 ± 3.50 | 9.52 ± 4.00 | 10.93 ± 4.07 | 2.16 | 0.117 |
| ​​Preoperative fasting duration for solids​, Mean ± SD | 10.20 ± 4.23 | 10.07 ± 3.73 | 10.01 ± 4.41 | 11.32 ± 4.47 | 1.63 | 0.199 |
| ​​PACU-to-ward transfer time​​, Mean ± SD | 1.10 ± 0.73 | 1.23 ± 0.77 | 1.01 ± 0.70 | 1.17 ± 0.71 | 2.95 | 0.054 |
| Surgery duration,  Mean ± SD | 3.87 ± 1.74 | 4.04 ± 1.72 | 3.78 ± 1.75 | 3.85 ± 1.74 | 0.70 | 0.499 |
| ​​VAS on admission, M (Q₁, Q₃) | 0.00 (0.00, 3.00) | 1.00 (0.00,3.00) | 0.00 (0.00,3.00) | 0.00 (0.00,2.25) | 1.04# | 0.595 |
| ​​VAS 30 minutes before surgery, M (Q₁, Q₃) | 0.00 (0.00, 0.00) | 0.00 (0.00,0.00) | 0.00 (0.00,0.00) | 0.00 (0.00,0.25) | 0.07# | 0.567 |
| VAS 2 hours postoperatively, M (Q₁, Q₃) | 2.00 (0.00, 3.00) | 2.00 (0.00,3.00) | 2.00 (0.00,3.00) | 0.00 (0.00,3.00) | 1.57# | 0.456 |
| ​VAS 6 hours postoperatively​​, M (Q₁, Q₃) | 0.00 (0.00, 2.00) | 0.00 (0.00,2.00) | 1.00 (0.00,2.00) | 0.00 (0.00,2.00) | 3.76# | 0.153 |
| Daily water intake, M (Q₁, Q₃) | 1100.00 (600.00, 1600.00) | 1000.00 (500.00,1600.00) | 1200.00 (735.00,1675.00) | 1000.00 (537.50,1300.00) | 3.22# | 0.200 |
| Preoperative fluid infusion, M (Q₁, Q₃) | 400.00 (200.00, 500.00) | 400.00 (300.00,512.50) | 400.00 (200.00,500.00) | 300.00 (200.00,500.00) | 7.30# | **0.026** |
| ​​Intraoperative total output​, M (Q₁, Q₃) | 400.00 (20.00, 900.00) | 400.00 (45.00,900.00) | 350.00 (20.00,950.00) | 375.00 (16.25,862.50) | 0.47# | 0.790 |
| ​​Intraoperative urine output​, M (Q₁, Q₃) | 300.00 (0.00, 700.00) | 250.00 (0.00,725.00) | 260.00 (0.00,700.00) | 300.00 (0.00,800.00) | 0.39# | 0.822 |
| ​​Intraoperative blood loss​, M (Q₁, Q₃) | 100.00 (15.00, 200.00) | 100.00 (20.00,300.00) | 50.00 (10.00,200.00) | 100.00 (0.00,162.50) | 3.25# | 0.197 |
| ​​Intraoperative fluid infusion volume​, M (Q₁, Q₃) | 1300.00 (800.00, 2100.00) | 1500.00 (900.00,2225.00) | 1300.00 (800.00,2100.00) | 1200.00 (700.00,1750.00) | 3.43# | 0.180 |
| ​​Intraoperative blood transfusion volume​, M (Q₁, Q₃) | 0.00 (0.00, 0.00) | 0.00 (0.00,0.00) | 0.00 (0.00,0.00) | 0.00 (0.00,0.00) | 0.75# | 0.688 |
| ​​postoperative 6-hour fluid infusion volume​, M (Q₁, Q₃) | 600.00 (100.00, 900.00) | 665.00 (200.00,1013.75) | 600.00 (100.00,800.00) | 600.00 (100.00,1000.00) | 5.46# | 0.065 |
| ​​postoperative 6-hour oral fluid intake​, M (Q₁, Q₃) | 300.00 (100.00, 500.00) | 300.00 (100.00,500.00) | 330.00 (150.00,500.00) | 200.00 (92.50,412.50) | 2.66# | 0.265 |
| ​​​postoperative 6-hour total output​, M (Q₁, Q₃) | 700.00 (475.00, 1000.00) | 700.00 (430.00,1000.00) | 700.00 (500.00,1000.00) | 700.00 (400.00,1177.50) | 0.10# | 0.951 |
| ​​postoperative 6-hour urine output​, M (Q₁, Q₃) | 675.00 (400.00, 1000.00) | 662.50 (400.00,918.75) | 700.00 (500.00,1000.00) | 665.00 (387.50,1150.00) | 0.20# | 0.905 |
| ​​postoperative 6-hour plasma drainage volume, M (Q₁, Q₃) | 0.00 (0.00, 80.00) | 0.00 (0.00,72.50) | 0.00 (0.00,80.00) | 0.00 (0.00,85.00) | 0.48# | 0.788 |

*F: ANOVA, #: Kruskal-waills test, χ²: Chi-square test, -: Fisher exact; SD: standard deviation, M: Median, Q₁: 1st Quartile, Q₃: 3st Quartile; PACU, Post-Anesthesia Care Unit; HADS-A, Hospital Anxiety and Depression Scale - Anxiety subscale​; HADS-D, Hospital Anxiety and Depression Scale - Depression subscale​; GCQ, General Comfort Questionnaire scores​; VAS, ​​Visual Analog Scale​;HEI, Huaxi Emotional Distress Index;​;PSQI, Pittsburgh Sleep Quality Index*
